# Antibiotic‐mediated bacteriome depletion in Apc^*Min/+*^ mice is associated with reduction in mucus‐producing goblet cells and increased colorectal cancer progression

**DOI:** 10.1002/cam4.1460

**Published:** 2018-04-06

**Authors:** Kamaljeet Kaur, Arpit Saxena, Irina Debnath, Jacqueline L. O'Brien, Nadim J. Ajami, Thomas A. Auchtung, Joseph F. Petrosino, Alexander‐Jacques Sougiannis, Sarah Depaep, Alexander Chumanevich, Phani M. Gummadidala, Mayomi H. Omebeyinje, Sourav Banerjee, Ioulia Chatzistamou, Paramita Chakraborty, Raja Fayad, Franklin G. Berger, James A. Carson, Anindya Chanda

**Affiliations:** ^1^ Exercise Science, Arnold School of Public Health University of South Carolina Columbia South Carolina; ^2^ Environmental Health Sciences, Arnold School of Public Health University of South Carolina Columbia South Carolina; ^3^ The Alkek Center for Metagenomics and Microbiome Research Department of Molecular Virology and Microbiology Baylor College of Medicine Houston Texas; ^4^ Mechanical Engineering University of South Carolina Columbia South Carolina; ^5^ Pathology, Microbiology& Immunology, School of Medicine University of South Carolina Columbia South Carolina; ^6^ Department of Statistics University of South Carolina Columbia South Carolina; ^7^ Center for Colon Cancer Research University of South Carolina Columbia South Carolina

**Keywords:** Antibiotics, Colorectal cancer, Goblet cells, Microbiome

## Abstract

Recent epidemiological evidence suggests that exposure to antibiotics in early‐to‐middle adulthood is associated with an increased risk of colorectal adenoma. However, mechanistic studies in established preclinical cancer to examine these claims are extremely limited. Therefore, we investigated the effect of long‐term exposure of an antibiotic cocktail composed of Vancomycin, Neomycin, and Streptomycin, on tumor development and progression in the *Apc* ^*Min/+*^ mouse, an established genetic model for familial adenomatous polyposis. Clinical pathologies related to tumor development as well as intestinal and colon tissue histopathology were studied at ages 8, 12, and 16 weeks of age, which correspond to the approximate ages of development of neoplasia, gut inflammation with polyposis, and cancer progression, respectively, in this animal model. We show that the antibiotics significantly increase the severity of clinical symptoms, including effects on intestinal histology and goblet cell numbers. In addition, they promote small intestinal polyposis. Finally, metagenomic analysis of fecal samples demonstrated that antibiotic exposure is associated with a significant but nonuniform depletion of the animal's natural gut flora. Overall, these findings support the premise that long‐term antibiotic exposure mediates the selected depletion of gut microbial communities and the concomitant thinning of the protective mucus layer, resulting in an increase in tumor development.

## Introduction

The gut microbiome is integral to gastrointestinal tract function and is connected to a variety of health issues 1, 2, 3. Its role has been demonstrated in a number of conditions, such as diabetes 4, metabolic disorders 5, Alzheimer's disease 6, systemic lupus erythematosus 7, hypertension 8, mental disorders 9, obesity 10, pancreatic disorders 11, cardiovascular disorders 12, aging 13, inflammatory disorders 1, and cancer 14, 15 including colorectal cancer 16.

Colorectal cancer is the second leading cause of cancer‐related death in the United States and ranks fourth in estimated new cases 17. The functional link between gut microbial dysbiosis and colorectal cancer is supported by preclinical studies with animal models 18, 19, 20, 21 and by clinical investigations with patients predisposed to colorectal cancer 22, 23, 24, 25, 26. Hence, the significant increase in the use of antibiotics among adults and children in the United States 27, 28, 29, 30, 31 is a public health concern. Accumulating evidence supports the notion that long‐term antibiotic exposure alters the functional capacity of the gut microbiota 32, 33 resulting in an increased risk of chronic gut diseases such as inflammatory bowel disease 34 and celiac disease 35 as well as activation of the biological mechanisms that initiate or promote colorectal carcinogenesis 36.

Despite these lines of evidence, there are significant gaps in our understanding of how antibiotics increase the risk of colorectal cancer. Current studies show that tetracycline mediates upregulation of cyclooxygenase‐2 and prostaglandin production 37, which promote chronic inflammation‐induced colorectal cancer 38. Addressing this knowledge gap is critical prior to clinical recommendations and development of microbial therapies to counter the effects of long‐term antibiotic use.

In the current study, we address this knowledge gap by examining the effects of long‐term administration of an antibiotic cocktail of Vancomycin, Neomycin, and Streptomycin on gut polyposis in the *Apc* ^*Min/+*^ mouse*,* an established genetic model for familial adenomatous polyposis 39. This model develops approximately 30–50 tumors in the gut at an age of 16–20 weeks with tumors mostly located toward the iliac part of the small intestine and the descending part of the colon 40. Reported here are our findings from the comparisons of clinical pathologies and the intestinal and colon tissue histopathologies related to colorectal cancer in antibiotic administered and control mice of at ages 8, 12, and 16 weeks. The ages, respectively, correspond to the approximate ages of neoplasia, gut inflammation with polyposis, and cancer progression in this animal model.

## Materials and Methods

### Experimental animal groups and diet

**Figure 1:**
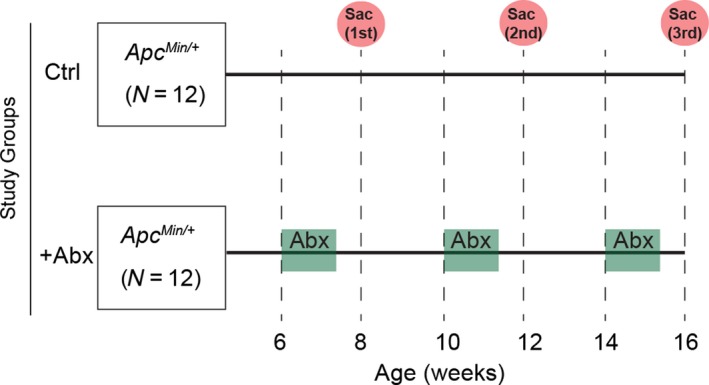
Study design. Two groups of *Apc* ^*Min/+*^ mice (*N* = 12 per group) were used for this study. Antibiotics were added in the drinking water of ‘+Abx mice’ at age 6, 10, and 14 weeks for a period of 10 days (green highlight). Clinical pathology, tissue histopathology, and polyposis in +Abx mice were compared with the control (Ctrl) mice, at 8, 12, and 16 week time‐points upon sacrificing four mice per time‐point (red circles).

*Apc* ^*Min/+*^ mice were obtained from Jackson Laboratories and bred in‐house at the Animal Resource Facility at the, University of South Carolina. Food (Purina chow) and drinking water were available to the mice *ad libidum* under a 12:12‐hour light–dark cycle and a low‐stress environment (22°C, 50% humidity, and low noise). At 4 weeks of age, littermates were randomly assigned to the following six groups (Fig. 1): (1) *Apc* ^*Min/+*^ untreated controls sacrificed after 8 weeks (2) *Apc* ^*Min/+*^ untreated controls sacrificed after 12 weeks, (3) *Apc* ^*Min/+*^ untreated controls sacrificed after 16 weeks, (4) *Apc* ^*Min/+*^ administered with antibiotics and sacrificed after 8 weeks, (5) *Apc* ^*Min/+*^ administered with antibiotics and sacrificed after 12 weeks and (6) *Apc* ^*Min/+*^ administered with antibiotics and sacrificed after 16 weeks. All procedures and animal care followed institutional guidelines and were approved by the Institutional Animal Care and Use Committee at the University of South Carolina. Four mice were assigned to each of the six treatment groups, and this sample size was based on statistical power analyses conducted in previous microbiome studies 41, 42, 43, which indicated that 3–5 mice is the required sample size for studying changes in the mice gut microbial communities following antibiotic treatment.

### Antibiotic administration

A mixture containing Vancomycin (1 mg/mL, active against Gram‐positive bacteria), Neomycin (1 mg/mL, active against Gram‐negative bacteria), and Ampicillin (1 mg/mL, active against both Gram‐positive and Gram‐negative bacteria) was administered to antibiotic treatment group (+Abx). The antibiotic mixture was added to the drinking water at age 6, 10, and 14 weeks (Fig. 1). Normal drinking water replaced the antibiotic containing water after 10 days of administration.

### Clinical score, histopathological assessments and polyp counts

The use of a ‘clinical score’ has been previously used to quantitatively express disease symptoms has been described previously 44, 45. The cumulative clinical score for each mouse, with a maximum score of 12, was based on weight loss measurement, diarrhea, and fecal hemoccult. There was a maximum score of four within each of the three quantitative parameters. Score for the weight loss was based on the following published scale where 0 = 0–5% weight loss; 1 = 6–10% weight loss; 2 = 11–15% weight loss; 3 = 16–20% weight loss; and 4 = >20% weight loss. Scoring of diarrhea was as follows: 0 = well‐formed pellets, 2 = pasty and semi‐formed stools that do not adhere to the anus, 4 = liquid stools that adhere to the anus. Detection of blood in the stools was determined using hemoccult kit (Beckman coulter, Brea, CA), which is a hydrogen peroxide‐based kit that forms a visible blue colored complex with blood. The followings were the score rates for the fecal hemoccult: 0 = no blood, 2 = positive hemoccult, 4 = gross bleeding. The total clinical score was the summation of the individual score of weight loss, diarrhea, and fecal hemoccult. Tumor quantification was conducted manually upon observing the 1% methyl blue stained tissue sections under the light microscope as described previously 44. Histopathological analyses for colonic tissue inflammation were conducted using a scoring system as described previously 44. Quantitative comparison of intestinal inflammation was conducted by comparisons of crypt depth‐to‐villus height ratio (CVR). Goblet‐to‐epithelial ratio per crypt was quantified upon analyses of intestinal tissue samples stained with alcian blue (for staining mucus‐containing goblet cells) and counterstained with Nuclear Fast Red solution (for staining the epithelial cells of the mucosa) as described previously [^44^].

### Fecal bacteriome analysis

Genomic DNA from fresh feces were isolated using the MoBio PowerSoil DNA Isolation Kit 46 and subjected to 16S rRNA gene analysis 47. The 16S metadata were demultiplexed with QIIME 48, 49. OTUs were shortlisted using OTUPipe analysis pipeline for error correction, chimera checking, UCLAST clustering and picking the optimal representative sequence centroid. Reference‐based chimera checking was conducted against a set of trusted sequences from the ‘Gold’ database 50. Taxonomy were assigned using the RDP classifier version 2.237 as described previously 51. The rendered OTU tables were checked for mislabeling and contamination as described previously 52. Finally, alpha‐diversity was estimated for each sample/sample pair using Chao1 (estimator of richness) and Shannon Diversity Index (richness and evenness). Also weighted UniFrac (dissimilarity based on phylogenetic differences and taxonomic abundance) and unweighted UniFrac (dissimilarity based on phylogenetic differences but not abundance) were used to express beta‐diversity 53 within and between the antibiotic‐administered and control mice.

### Statistical analysis

Two‐way analysis of variance (ANOVA), two‐way repeated‐measure ANOVA, and one‐way ANOVA were used to analyze the data. A Tukey post hoc analysis was used to determine differences in physiological responses upon antibiotic‐administered mice and the controls. All statistical analyses were performed with SigmaStat 3.5 (SPSS, Chicago, IL). For fecal bacteriome analyses, Kruskal–Wallis and Mann–Whitney statistical analyses were performed to calculate significance in diversity and relative abundance, respectively. A *P* value of <0.05 was considered significant.

## Results

### Effect of antibiotic administration on total polyp counts and clinical pathology

**Figure 2:**
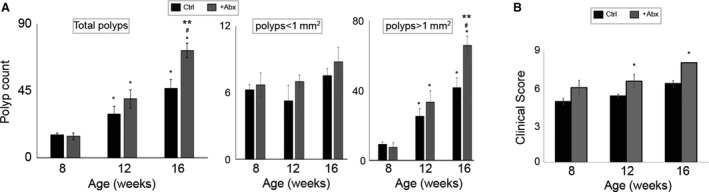
Effect of antibiotic exposure on polyposis and clinical scores: (A) Polyp counts. Graph representing the small intestine polyp in Control (Ctrl) and + Abx mice; *left panel,* total count, *middle panel*, count of polyps of size < 1 mm^2^, *right panel*, count of polyps of size >1 mm^2^. Two‐way repeated‐measure analysis of variance (ANOVA) was applied to calculate the significant difference between the polyp counts between +Abx and Ctrl groups at the different ages (**P < 0.01 Ctrl vs. +Abx within each time‐point, *P < 0.05 vs. 8 weeks in each group, #*P* < 0.05 16 weeks vs. 12 weeks in each group), (B) Clinical score. Comparison of clinical scores for +Abx and Ctrl groups at different time‐points during the study. Two‐way repeated‐measure analysis of variance (ANOVA) was applied to calculate the significant difference between the clinical scores between +Abx and Ctrl groups (*P < 0.05 +Abx vs. Ctrl).

To mimic long‐term antibiotic exposure, *Apc* ^*Min/+*^ mice were exposed to the antibiotic cocktail beginning at 6, 10, and 14 weeks of age, and polyp numbers and sizes were assessed at 8, 12, and 16 weeks (Fig. 1). As shown in Figure 2A, total polyp counts were significantly higher for antibiotic‐administered mice at 12 and 16 weeks, as compared to control mice. This was predominantly due to larger polyps (i.e., ≥1 mm^2^), as there were no significant differences for polyps <1 mm^2^. Thus, antibiotic exposure promoted development of intestinal polyps.

As shown in Figure 2B, clinical scores correlated with the polyp counts in that antibiotic‐administered mice demonstrated significantly higher scores compared to the controls at 12 and 16 weeks of age.

### Effect of antibiotic administration on intestinal histopathological scores and crypt‐to‐villus ratios

**Figure 3:**
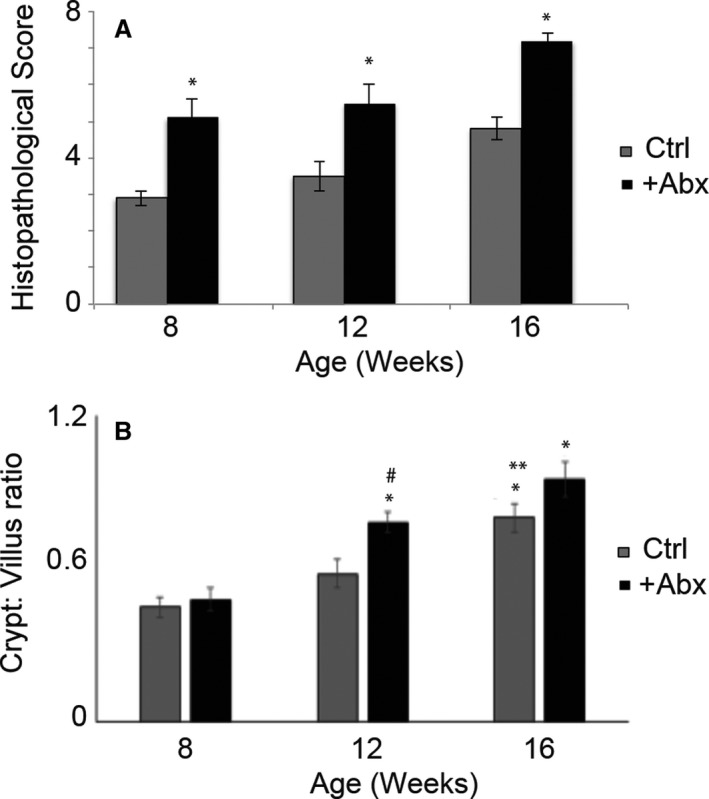
Analysis of tissue inflammation. (A) Histopathology scoring. The scoring was based on the analysis of 10 different sections determining the degree of inflammation and immune cell infiltration was plotted for Ctrl and +Abx groups at different ages. Two‐way analysis of variance (ANOVA) was used to calculate the significant difference between groups at different ages (*P < 0.05‐ +Abx vs. Ctrl). (B) Intestinal CVR. CVR was calculated as a measure of intestinal inflammation for all animals. Two‐way analysis of variance (ANOVA) was used to determine the significance of difference between Ctrl and +Abx mice at different ages. *P value<0.04 versus 8 weeks, ^#^ *P* < 0.01 +Abx versus Ctrl and **P < 0.05 16 versus 12 weeks within the same group.

Clinical scores derived from an integral assessment of weight loss, diarrhea, and fecal hemoccult were observed to increase in antibiotic administered as compared to control mice at all three ages (Fig. 3A; a representation of the H&E stained tissues is shown in Fig. S1). Crypt‐to‐villus ratios were higher in antibiotic treated as compared to control mice at 12 and 16, but not 8, weeks (Fig. 3B). These results suggest an increase in intestinal and colonic inflammation upon long‐term antibiotic administration.

### Effects on goblet cell counts

**Figure 4:**
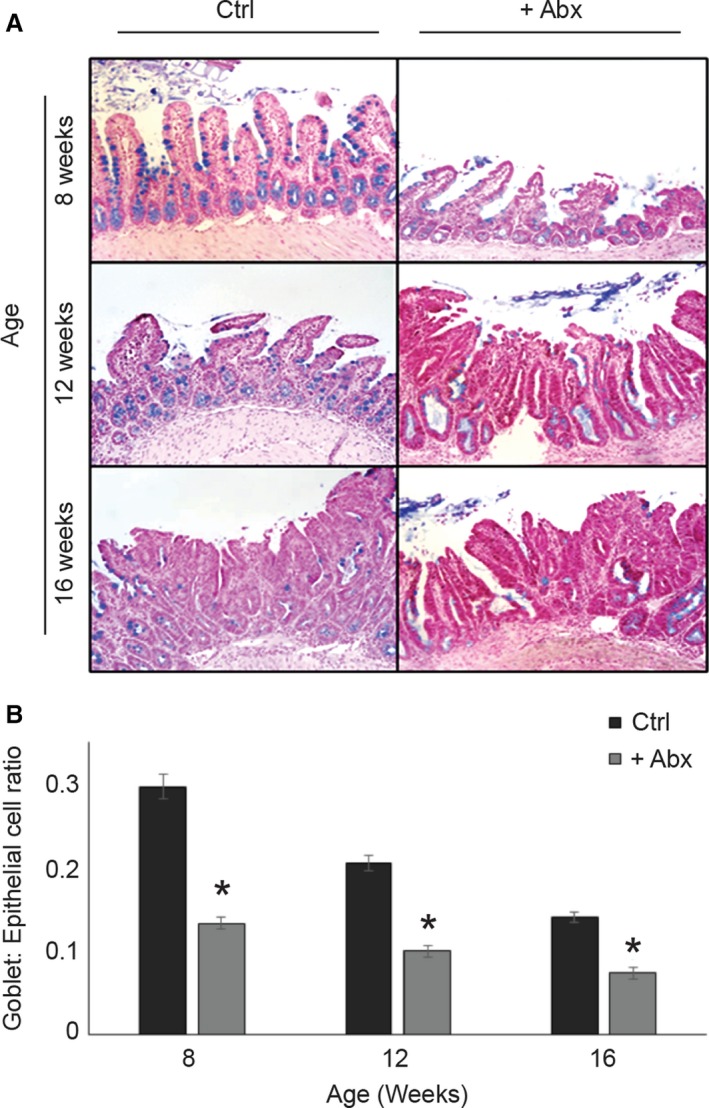
Effect of antibiotic exposure on goblet cell development. (A) Representative Alcian blue stained tissue sections from Ctrl and +Abx mice. Blue and pink staining indicates goblet and epithelial cells, respectively. (B) Graph showing goblet‐to‐epithelial cell ratio per view per group. Two‐way ANOVA was used to determine the significant difference in goblet to epithelial cell ratio between groups. *P < 0.01 Ctrl versus +Abx group.

As antibiotics impact the thickness of the intestinal mucus layer 54, we investigated the effects of the antibiotic treatment on the numbers of mucus‐producing goblet cells. Results (Fig. 4) indicated a significant drop in the ratio of goblet to epithelial cells in mice exposed to antibiotics at all three ages. These effects may be due to either an increase in goblet cell apoptosis or a decrease in production.

### Effect of antibiotics on the fecal bacteriome

**Figure 5:**
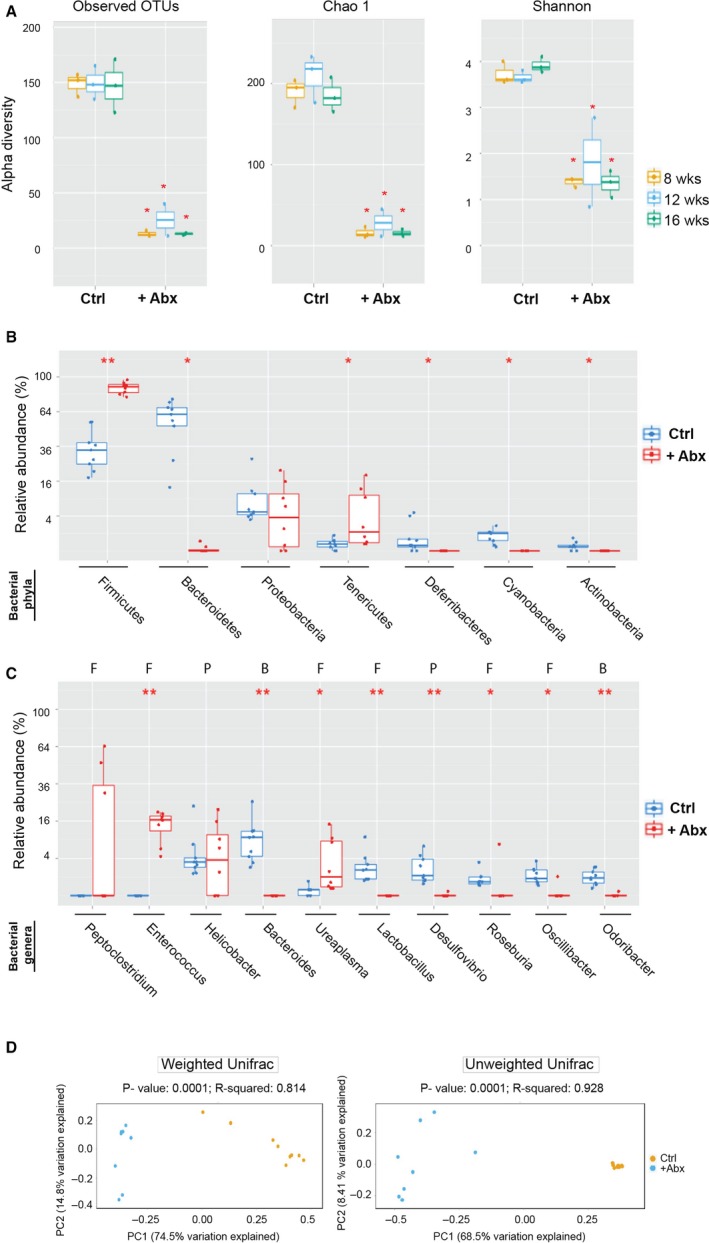
Effect of antibiotic administration on fecal bacteriome. (A) Alpha‐diversity measures in fecal samples. Depletion of fecal bacteriome in +Abx mice was demonstrated using observed OTUs and Chao1 diversity measures and depletion of bacteriome richness was demonstrated using Shannon diversity measures. Comparisons to determine age‐dependent variance were performed using Kruskal–Wallis analyses of variance. *P*‐values determined from these analyses for OTU, Chao, and Shannon indices were 0.96, 0.29, and 0.29, respectively, for Ctrl mice and 0.5, 0.3, and 0.97, respectively, for +Abx mice. *P*‐values to assess significance in change of alpha‐diversity indices were determined using Mann–Whitney *U*‐test, (*) *P* < 0.05, +Abx versus Ctrl for all age groups. (B) Relative abundance of top seven predominant bacterial phyla in Ctrl and +Abx mice. Statistical significance was assessed by Mann–Whitney *U*‐test (*) represents *P *< 0.05, (**) represents *P *≤ 0.0001. (C) Relative abundance of top seven bacterial genera in Ctrl and +Abx mice. F, genus belongs to phylum Firmicutes, P, genus belongs to phylum Proteobacter, B, genus belongs to phylum Bacteroidetes. Statistical significance was assessed by Mann–Whitney *U*‐test (*) represents *P* < 0.05, (**) represents *P *< 0.001. As panel A demonstrated that alpha‐diversity was age independent, the 16S rRNA reads from mice of all time‐points were pooled for analysis while comparing Ctrl and +Abx mice in panels B and C. (D) Weighted and unweighted beta‐diversity for Ctrl and +Abx fecal samples. Plots of PCoA based on weighted and unweighted UniFrac distance matrices of microbial communities in fecal samples of Ctrl and +Abx mice. For weighted and unweighted beta‐diversity, PC1 (*x*‐axis) explained 74.5% and 68.5% of variability, respectively, and PC2 (*y*‐axis) explained 14.8% and 8.4% of variability, respectively.

The composition of the fecal bacteriome was compared between control and antibiotic‐administered mice. As shown in Figure 5A, alpha‐diversity did not change significantly with age in either the control or the antibiotic exposed mice, suggesting that *Apc* ^*Min/+*^ mice retained a stable microbiome during the time window of 8–16 weeks. However, for all age groups, alpha‐diversity was significantly decreased by antibiotic treatment, as measured by total operational taxonomic units (OTUs), the Chao1 index, or the Shannon index (Fig. 5A). Similar decreases were observed in 8, 12, and 16 weeks.

As age had no significant effect on alpha‐diversity of either control or treated groups, we pooled the 16S rRNA reads from all the time‐points to determine the predominant phyla within each group. For both control and treated mice, the 16S rRNA reads were assigned to seven phyla, of which *Bacteroidetes*,* Firmicutes,* and *Proteobacteria* constituted were most predominant. As shown in Figure 5B, *Bacteroidetes* and *Firmicutes* showed significant shifts in relative abundance upon antibiotic exposure. Abundance for *Bacteriodetes* decreased from 60% to <5%, while that for *Firmicutes increased by* nearly threefold.

Alterations at the genus level were also assessed (Fig. 5C). While a significant elevation in abundance of three *Firmicutes* genera (*Enterococcus*,* Ureaplasma,* and *Peptoclostridium*) was measured in response to antibiotic treatment, several probiotic genera (*Bacteroides, Lactobacillus, Desulfovibrio*, among others) were nearly eliminated. The shifts in bacterial abundance upon antibiotic administration were also reflected by the beta‐diversity patterns (Fig. 5D), in that significant differences were observed at all ages between the antibiotic‐administered and control groups in both unweighted and weighted UniFrac distance metrics (*P* = 0.0001).

Overall, the results demonstrate that administration of antibiotics eliminated the majority of bacterial flora, with the most drastic depletion being observed for the phylum *Bacteroides* and several beneficial genera within *Firmicutes*.

## Discussion

In the current study, we provide direct experimental evidence in support of the notion that long‐term exposure to antibiotics can promote polyp development in the gut of a genetically susceptible mouse strain, as suggested by previous epidemiological reports that linked colorectal carcinogenesis to antibiotic use in humans 36, 55, 56, 57, 58. We emphasize that the significance of our findings expands beyond colorectal cancer, as a number of recent studies have shown that disruption of the innate microbiota by low‐dose antibiotic exposures, even if limited to transient perturbations early in life, can have long‐term metabolic alterations and affect ileal expression of genes involved in immunity 59. Recent reports from Boursi et al. 60. also indicate that antibiotic exposures increase the risk of diabetes, which in turn increases the risk of developing colorectal cancer 61, 62, as well as lung, prostrate, gastric, and breast cancers 63.

An intriguing and novel aspect of the current study is the observation that depletion of the natural bacterial flora upon antibiotic exposure correlates with reduction in mucus‐producing goblet cell numbers, along with an increase in both colon histopathological scores and intestinal crypt‐to‐villus ratios. The functional link between antibiotic‐mediated microbial dysbiosis and the inhibitory effects of antibiotics on development of goblet cells is of particular interest, as these cells are integral to protection against inflammation and polyposis. In all, this is consistent with previous findings suggesting that gut microbiota impact the thickness of the mucus layer 54, which provides nutrition and energy to the intestinal microflora by enabling them to break down and utilize the glycans present in the mucus 64.

**Figure 6:**
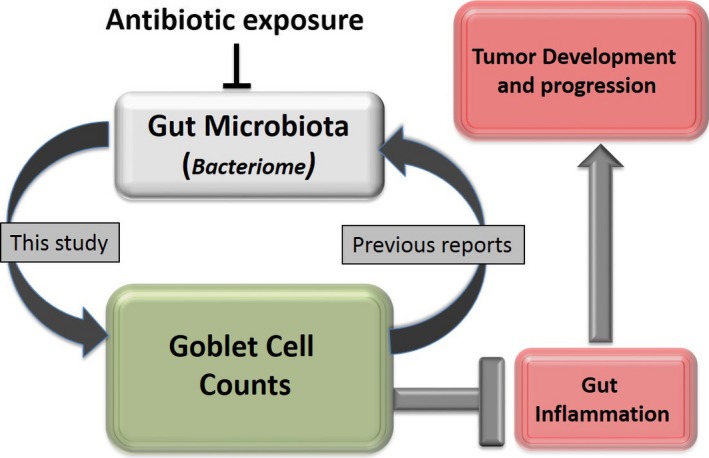
Microbiome‐goblet cell protection model. According to this model, the composition of the natural gut bacterial flora is associated with gut mucosal goblet cell counts. Depletion of bacterial communities reduced goblet counts that could be attributed to either regulation of goblet cell apoptosis or goblet cell development or both. Goblet cells are integral to the maintenance of the mucus layer, which in turn, regulates the composition of the gut microflora (findings from previous studies 64). As mucus layer offer protection against inflammation and tumor progression 44, factors that disrupts this protection cycle will lead to gut inflammation and carcinogenesis.

Our results imply that the intestinal microbiome has a profound impact on the global physical properties of the gut, which in turn determines the severity of inflammation and cancer. This is illustrated by our ‘cyclical microbiome protection model’ depicted in Figure 6. According to this model, factors such as long‐term antibiotic exposures lead to gut microbial dysbiosis and decrease goblet cell counts. This in turn increases the severity of microbial dysbiosis, eventually leading to inflammation and cancer.

The choice of the antibiotics for this study was based on their known mechanisms of action in bacterial cells and their minimal nontoxic effects on the host. Neomycin is used against both Gram‐positive and Gram‐negative bacteria. To minimize nephrotoxic effects of the antibiotic, it is prescribed as an oral antibiotic. Its inhibits bacterial growth by binding RNA duplexes 65 and is water soluble with a low toxicity in animals 66. Vancomycin, which inhibits Gram‐positive bacteria 67, 68, is not absorbed by the intestinal mucosa 69 and acts by inhibiting cell wall formation through binding to the terminal D‐alanyl‐D‐alanine moieties of the N‐acetyl glucosamine/N‐acetyl muramic acid peptide 70. Ampicillin is active against both Gram‐positive and Gram‐negative bacteria and is typically nontoxic 71. It penetrates bacterial cell walls and irreversibly inhibits the enzyme transpeptidase that is needed for cell wall synthesis, thereby leading to cell lysis 71. Based on a plethora of evidence on direct immunomodulatory effects of antibiotics 72, we cannot rule out the notion that the antibiotics used in this study, in addition to having antibacterial effects, act directly on the physiology of the animal host. However such alternate possibilities do not undermine the primary conclusions of the current study.

Interestingly, the antibiotic cocktail used currently selectively depleted the genus *Bacteroides* and some ‘beneficial’ genera such as *Lactobacillus* and *Desulfovibrio*. This implicates the possible role of these genera in protection against cancer progression. The increase in larger polyps during cancer progression may be due to selective depletion of these bacterial genera. Alternatively, the dramatic increase in relative abundance of *Firmicutes* upon antibiotic administration may underlie increased polyposis. Distinguishing between these and other possibilities will require follow‐up studies with gnotobiotic rodent models. Our findings are different from observations described by Hamoya et al. 73., in which low‐dose erythromycin exposure (0.5 mg/mL) reduced polyposis in *Apc* ^*Min/+*^ mice. It may be that different antibiotic exposures may result in distinct microbial profiles, which in turn, may lead to variation in disease outcomes.

Our follow‐up studies will investigate the effect of these potential ‘therapeutic microbes’ on cancer development and progression. Other aspects to be investigated in our follow‐up studies are use of different antibiotics, and the timing of antibiotic exposures relative to polyp development. Such studies will provide additional mechanistic information on the association of antibiotic exposure, effects on the gut microbiome, and development of colorectal carcinogenesis.

## Conflict of Interest

The authors declare no conflict of interest.

## Supporting information


**Figure S1**. Representative Hemeatoxylin and eosin (H&E) stained colon tissues belonging to control and +Abx mice at different ages.
