# Supplementary figures and images for: Antibiotic‐mediated bacteriome depletion in ApcMin/+ mice is associated with reduction in mucus‐producing goblet cells and increased colorectal cancer progression
Source: Cancer Med. 2018 Apr 6;7(5):2003–12. doi: 10.1002/cam4.1460 (PMC5943478; doi:10.1002/cam4.1460)

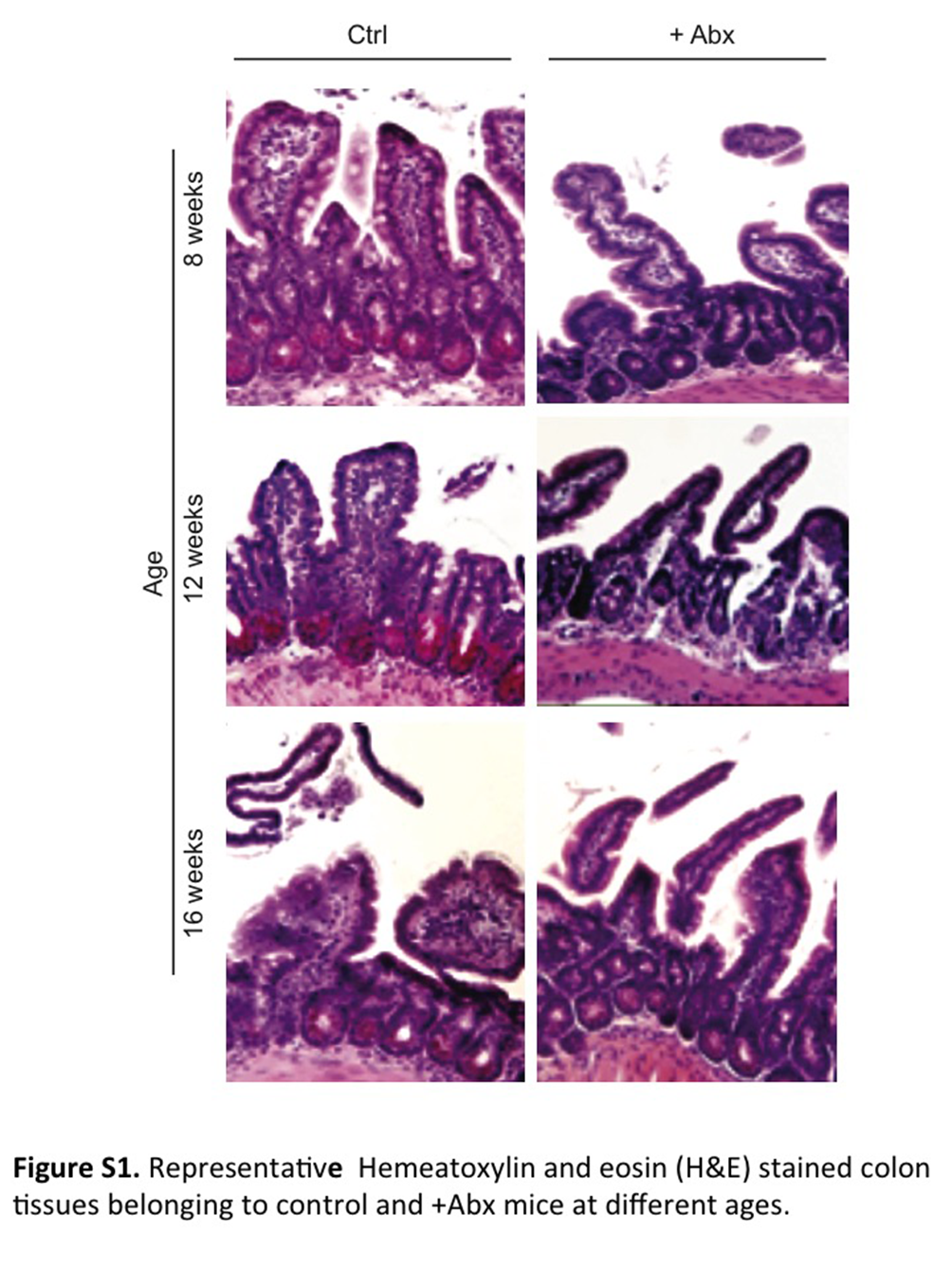

Supplement: Supplementary file 1 — Figure S1. Representative Hemeatoxylin and eosin (H&E) stained colon tissues belonging to control and +Abx mice at different ages. [file CAM4-7-2003-s001.tif]
